# Corrigendum to: Regional Differences in Brain-Derived Neurotrophic Factor Levels and Dendritic Spine Density Confer Resilience to Inescapable Stress

**DOI:** 10.1093/ijnp/pyab018

**Published:** 2021-05-03

Yang, C., Shirayama, Y., Zhang, J-C., Ren, Q., and Hashimoto, K. (2015) Regional differences in brain-derived neurotrophic factor levels and dendritic spine density confer resilience to inescapable stress. *Journal of International Neuropsychopharmacology*, 18(7):pyu121.

A reader noticed that Golgi staining in Figure 2d (control group) and 2h (LH group) were identical. In addition, Golgi staining in Figure 2d (LH group) and 2h (control group) were identical. The authors confirmed that there was an accidental duplicate of Golgi staining in Figure 2d and 2h. We corrected to the new Figure 2h (control, non-LH, and LH group). The authors declared that these errors in Golgi staining are unintentional. The corrected version of Figure 2 has been provided and replaced.

**Figure 2.**
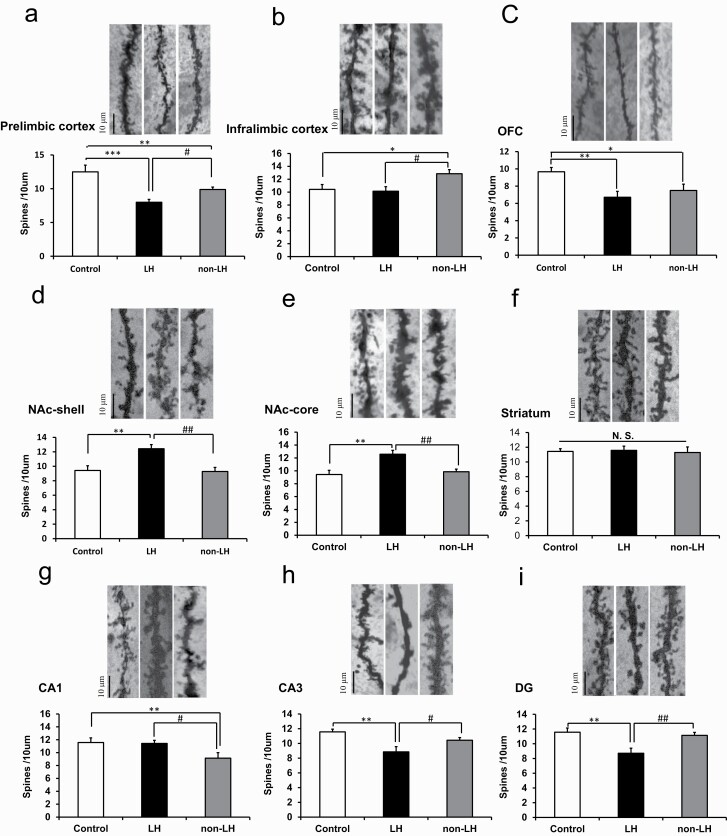
Spine densities in the brain regions of control, learned helplessness (LH), and non-LH groups. Golgi staining in the rat brains of control (n = 6), LH (n = 7), and non-LH (n= 7) groups was performed. Spine density in the prelimbic cortex, infralimbic cortex, OFC (orbitofrontal cortex), nucleus accumbens (NAc)-shell, NAc-core, striatum, CA1, CA3, and DG (dentate gyrus) of the hippocampus was measured. Data are shown as mean ± standard error of the mean. *p < 0.05, **p < 0.01, ***p < 0.001 compared to control group. ^#^*p* < 0.05, ^##^*p* < 0.01 compared to LH group. The bar is 10 μm.

